# Effect of Atmospheric Conditions on LIBS Spectra

**DOI:** 10.3390/s100504907

**Published:** 2010-05-14

**Authors:** Andrew J. Effenberger, Jill R. Scott

**Affiliations:** Idaho National Laboratory (INL), 1765 W. Yellowstone HWY, Idaho Falls, ID 83415-2208, USA; E-Mail: andrew.effenberger@inl.gov (A.J.E.)

**Keywords:** LIBS, vacuum, pressure, He, Ar, CO_2_, resolution, isotopes, Mars

## Abstract

Laser-induced breakdown spectroscopy (LIBS) is typically performed at ambient Earth atmospheric conditions. However, interest in LIBS in other atmospheric conditions has increased in recent years, especially for use in space exploration (e.g., Mars and Lunar) or to improve resolution for isotopic signatures. This review focuses on what has been reported about the performance of LIBS in reduced pressure environments as well as in various gases other than air.

## 1. Introduction

Laser-induced breakdown spectroscopy (LIBS) is a popular technique because of its speed, simplicity, and usually inexpensive hardware. Additionally, LIBS requires little or no sample preparation and can provide simultaneous multi-element analysis. Thus, it is not surprising that LIBS has been used for a wide variety of applications, such as material analysis [1], environmental monitoring [2–4], forensics [5], biological identification [4,6], and even characterization of fossils [7] and works of art [8]. There are several excellent reviews [9,10] and books [11–13] on LIBS and its applications. Typically, these applications occur under standard Earth atmospheric conditions (*i.e.*, 760 Torr). However, interest in LIBS under other atmospheric conditions has been a growing area of study both for fundamental knowledge and challenging applications.

**Figure 1.**
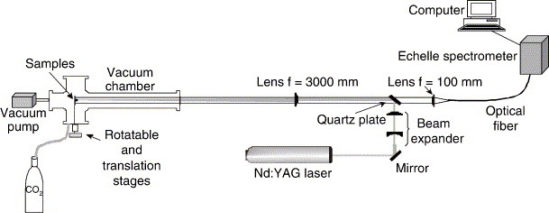
Schematic of typical apparatus for a pressure and gas composition LIBS studies. Reprinted from reference [23].

LIBS in pressures and atmospheric compositions other than Earth ambient has gained interest as LIBS has been promoted for space exploration applications [14–16]. ChemCam [17] is a remote LIBS instrument designed for the Mars Science Laboratory (MSL) rover nicknamed “Curiosity”, scheduled for launch in 2011. The potential of LIBS for exploration on Mars, which has an atmosphere with a pressure of ∼7 Torr composed of ∼95% CO_2_, has been explored primarily for geological characterization [18–26]. Currently, LIBS is also being explored for future Lunar applications [16,27,28], which is highly challenging because the lack of atmosphere on the moon means that the LIBS plasma would need to be observed in vacuum. In contrast, LIBS is also being explored for high pressure space environments such as Venus [29]. Determining isotope ratios is another application that has lead to the use of LIBS in reduced pressure environment because of the increased need for high resolution spectra [30–33]. Besides the usual LIBS equipment, these pressure/atmospheric studies also require a chamber to control the atmospheric conditions, which include ablation chambers equipped with vacuum pumps and gauges for controlling the pressure as well as gas inlets and mixing manifolds for varying buffer gases (Figure 1) [34].

While there have been several studies of LIBS under non-Earth ambient conditions, none of the studies currently available are comprehensive. Therefore, this review focuses on compiling an understanding of LIBS phenomena that have been gained through the various pressure dependence and atmospheric composition studies. The pressure studies have been divided into two regimes: >760 Torr and <760 Torr. The gas composition studies include comparisons of air, He, N_2_, Ar, and CO_2_, which may also report results incorporating pressure dependent experiments. Studies examining the effect of pressure conditions on LIBS of ambient gas have been investigated [35], however, the scope of this review will focus on the effect on ambient conditions on LIBS spectra generated from surfaces.

## 2. Influence of Pressure

### 2.1. Low Pressure, <760 Torr

Performing LIBS on a surface at reduced pressures (pressures below atmosphere) can result in enhanced spectra and improved ablation. Specifically, these enhancements are an increase in spectral intensity, spectra signal-to-noise (S/N), spectra resolution, increased ablation, and more uniformed ablation craters. These enhancements are generally seen when using both femtosecond and nanosecond lasers; however, the explanation for these enhancements vary slightly for the two laser’s pulsewidths.

**Figure 2.**
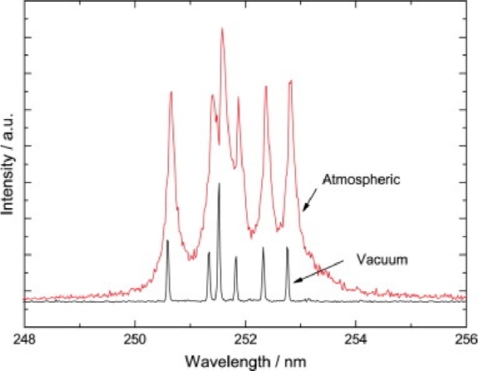
Comparison of LIBS spectra of Si at atmospheric and 10-6 Torr. Reprinted from reference [38].

In demonstrating the efficacy of a fiber optic feed-through design for integration of LIBS in a vacuum environment, Cowpe and Pilkington [36] produced high quality LIBS spectra at very low pressures. The experiments were carried out using a laser pulse from a Nd:YAG operating in the second harmonic (532 nm) with a 4–6 ns pulse duration through a fiber bundle while also collecting the LIBS spectra through the same bundle. Figure 2 compares LIBS spectra taken at atmospheric condition and at vacuum conditions, ∼10^−5^ Torr. Though the intensity of the LIBS spectrum taken at vacuum is less intense than the LIBS spectrum taken at atmospheric condition, it is clear that the LIBS spectrum at vacuum is of higher resolution [37,38]. This improved resolution is likely a result of the decreased electron density, which is also confirmed in work by Dreyer *et al*. [39].

**Figure 3.**
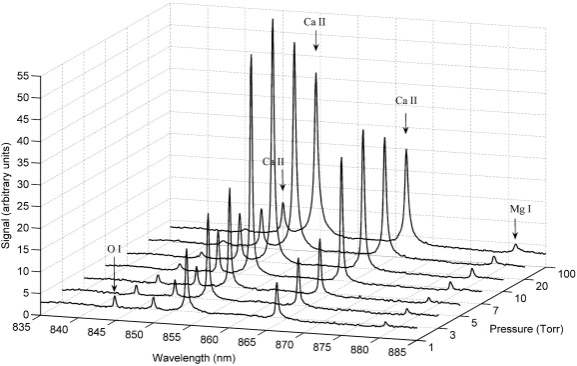
LIBS spectra of a geological sample (oolithic hematite) at various pressures. Reprinted from reference [39].

Using a Nd:YLF laser with a 10 ns pulse duration, Dreyer *et al*. performed LIBS at varying reduced pressures on a hematite sample [39]. Figure 3 shows several LIBS spectra at varying pressures, focusing primarily on Ca and Mg lines. It can be observed in Figure 3 that as the surrounding pressure is decreased from 100 mbar to 10 mbar, a gradual increase in LIBS intensity is observed. Also, a maximum intensity occurs between 5 and 10 Torr, while pressures below 5 Torr resulted in a significant decrease in LIBS emission intensity. This significant decrease in emission intensity at very low pressures is also seen in work by Shu *et al*. comparing LIBS at lunar simulated condition of 5 × 10^−5^ Pa (∼10^−7^ Torr) and atmospheric conditions [16]. Dreyer *et al*. also observe a significant decrease in spectral intensity of ionic species between 7 and 5 Torr, suggesting a rapid decrease in electron density [39]. Dreyer *et al*. analyzed the LIBS spectra using a non-gated spectrometer. It is well known that a gated spectrometer yields higher quality LIBS spectra if the time is optimized; however, Dreyer *et al*. chose a non-gated spectrometer to remove some bias introduced in gated schemes when timing is optimized for one condition and is then carried through for all other conditions, which is a conundrum that other researchers have observed and dealt with in various ways.

In addition to having an effect on the emission intensity and resolution, reduced pressures have also been shown to effect ablation from LIBS significantly. For example, in work performed by Vadillo *et al*. [40], the ablation from a laser plasma generated with a dye laser pumped by a XeCl laser with a 28 ns pulse width on iron or zinc samples showed a rather significant increase at 0.75 Torr compared to 750 Torr. At a laser fluence of 10 J/cm^−2^, Fe showed a 2.2-fold increase in ablation rate in 0.75 Torr Ar compared to 750 Torr Ar atmosphere. A similar result was seen on ablation of Zn. With a laser fluence of approximately 3 J/cm^−2^, the Zn ablation rate was 3.7-fold greater in 0.75 Torr Ar compared to 750 Torr Ar atmosphere. It was also noted by Vadillo *et al*. that the crater rims were free of deposited material after ablation at 0.75 Torr, while ablation at 750 Torr left craters with a visible ring of deposited material [40]. In other work by Vadillo *et al*., the 498.17 nm emission line from Ti(I) was monitored during laser ablation studies [41]. It was found that the emission intensity decreased with decreasing pressure. This result differs from other findings but this is likely due to the difference in which the experiments were conducted. Vadillo *et al*. [41] made the spectral measurements during ablation at a single location on the sample, while others avoided repeated ablation at the same location or at least minimized multiple shots at the same location to fewer than 40 shots. Of course continuous ablation at the same location will lead to deep craters and these craters will affect LIBS intensity. Some studies have shown that LIBS in a confined location, for example ablation craters, has a significant effect on the signal intensity [42–44]. Dreyer *et al*. noted reduced LIBS intensity after 10 to 20 shots at the same location [39].

**Figure 4.**
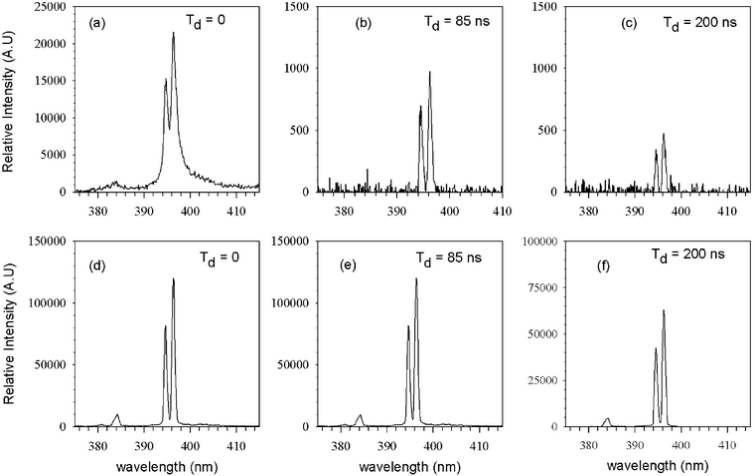
LIBS spectra of Al at atmospheric conditions **(a–c)** and at 4 Torr **(d–f)** with increasing delay times (0, 85 and 200 ns). Reprinted from reference [45].

**Figure 5.**
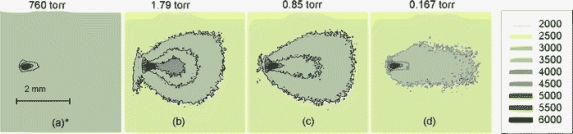
Two-dimensional Cu plasma images at different pressures produced using an accumulation of ten 50 μJ laser pulses, 10 ns second delay and 500 ns gate width. Reprinted from reference [45].

Yalcin and co-workers [45] investigated the effect of reduced pressures on LIBS using a Ti:sapphire laser with a 130 fs pulse duration. Figure 4 compares LIBS spectra of Al(I) at 396.15 nm taken at atmospheric pressure (760 Torr) and 4 Torr with spectrometer gate delays of 0, 85 and 200 ns, while maintaining a gate width of 100 ns. There is significant spectral enhancement at 4 Torr compared to atmospheric conditions at all gate delays. Yalcin *et al*. reported an S/N enhancement at 4 Torr compared to atmospheric condition of 240-, 840-, and 629-fold for 0, 85, and 200 ns delays, respectively. It was also observed that a Mg(I) line at 383.8 nm, which was nearly unresolved at atmospheric condition, is easily resolved at 4 Torr. As pressure is reduced further, much of the enhancement seen at 4 Torr is lost, which can be explained by Figure 5. Here Yalcin *et al*. examined 2D plasma images of a Cu plasma at 760, 1.79, 0.85, and 0.167 Torr. Figure 5 illustrates that the plasma becomes less ordered with decreasing pressures and expands more toward the laser as pressure is reduced. Yalcin *et al*. explains that this expansion causes a decrease in collision excitation, resulting in a dimmer (*i.e.*, less intense) plasma. The enhancement seen at 4 Torr compared to atmosphere is likely a result of reduced cooling of the plasma at low pressures. Yalcin and co-workers showed that the lifetime of a LIBS plasma created at 4 Torr is much greater than that of a LIBS plasma created at atmospheric condition. Because of this increased lifetime, the LIBS plasma emission is stronger for a longer period of time, which results in enhancement. There was no significant difference in ablation at 4 Torr and atmospheric pressure, which may be unique to LIBS plasma generated with femtosecond lasers [46].

Whether a femtosecond or nanoscecond laser is employed in surface LIBS experiments, there are some advantages in lowering the surrounding pressure. These advantages are higher spectral resolution, greater S/N, and increased spectral intensity. For ablation, it appears that only lasers with nanosecond pulse lengths can take advantage of lower pressures. The majority of photons from femtosecond lasers reach the surface before the laser plasma develops, while with the nanosecond pulse lasers, a significant portion of the photons are able to interact with the expanding plume. At reduced pressures, a plasma generated with a nanosecond pulse is expanding in a less dense atmosphere, which results in a less dense shock wave. The reduced density in the shock wave results in reduced plasma shielding; thus, allowing more photons to reach the sample. Increasing the number of photon interacting with surface results in increased sample ablation, which can also lead to a more intense spectrum. Because there is little plasma shielding during LIBS using a femtosecond laser, another explanation must be considered for the spectral enhancement observed at reduced pressures. During LIBS plasma expansion, energy is lost to the surrounding atmosphere. This loss of energy reduces the lifetime of the laser plasma. Therefore, reducing the pressure increases the lifetime of the plasma, allowing for more light from the laser plasma to be collected. If pressures are too low (<∼7 Torr), there is a steep loss in LIBS spectral intensity. This loss in intensity is likely due to a disordered plasma, which results from the lack of sufficient atmosphere to provide adequate confinement.

### 2.2. High Pressure, >760 Torr

**Figure 6.**
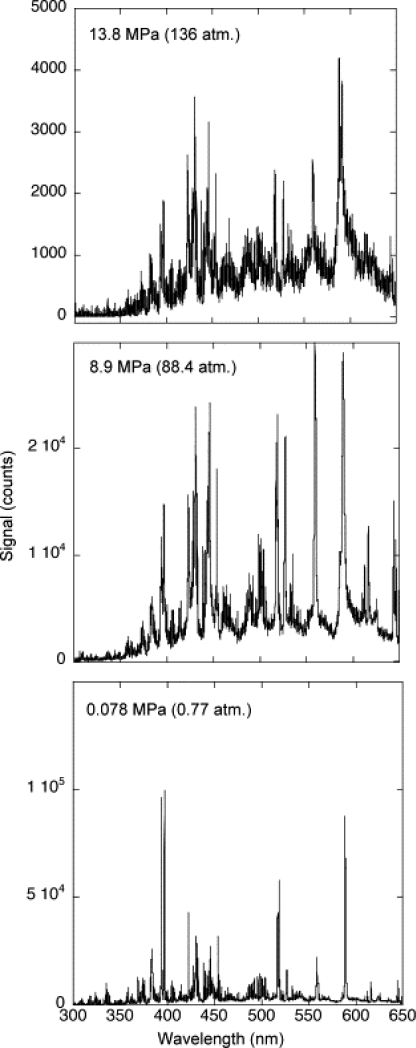
LIBS spectra of basalt taken in 0.77, 88.4 and 136 atm. Reprinted from reference [29].

LIBS at high pressure has been primarily investigated for applications. For example, Arp *et al*. investigated the effect of background high-pressures on LIBS of Basalt rock to examine the potential use of LIBS for future Venus missions [29]. Figure 6 shows the degradation of LIBS spectra over a pressure range of 0.77 to 136 atm (590 to 10^5^ Torr, respectively). Figure 6 clearly shows that as pressure increases, the LIBS intensity and S/N are greatly reduced. Under closer examination using a higher resolution spectrometer, spectra show significant peak broadening and self-absorption as pressures increased. Arp *et al*. [29] noted that it may be possible the plasma and or the laser pulse could have been defocused or misaligned due to the increase of pressure, resulting in reduced intensity and S/N.

**Figure 7.**
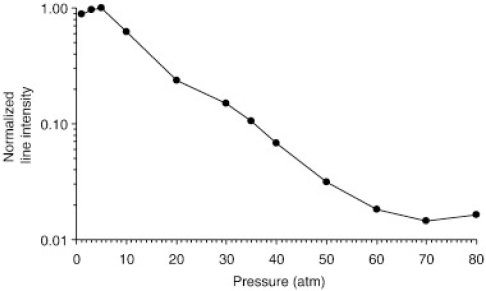
LIBS spectra intensity of C *versus* an increasing He atmosphere. Reprinted from reference [47].

**Figure 8.**
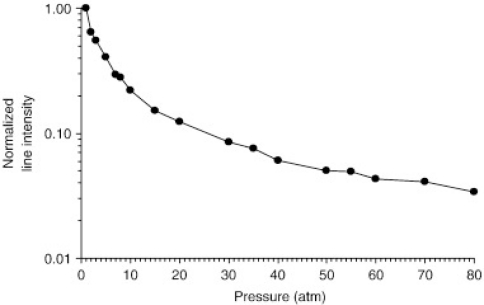
LIBS spectra intensity of C *versus* an increasing N_2_ atmosphere. Reprinted from reference [47].

Vors *et al*. studied the influence of increasing atmospheric pressure (1 to 80 atm) of N_2_ and He on LIBS spectra of carbon [47]. The high-pressure environment serves to replicate conditions in a nuclear reactor. LIBS spectra of C taken in a He atmosphere of 1 atm resulted in spectral intensity of at least 3 times greater than LIBS spectra in a N_2_ atmosphere at 1 atm. Vors *et al*. found that LIBS spectral intensity decreases with increasing pressures when either He or N_2_ are used. Interestingly, LIBS spectral intensity decreased more rapidly with increasing He atmospheric pressure than with increasing N_2_ pressure, which can be seen when comparing Figures 7 and 8. Vors *et al*. argues that this more rapid decrease in LIBS spectral intensity is likely due to the greater thermal conductivity of He compared to N_2_. The higher the thermal conductivity, the more rapidly a LIBS plasma will cool, which leads to a shorter plasma lifetime.

In a study examining Al(II) at 281.6 nm and Al(I) at 308.2 nm lines from LIBS, Owens and Majidi [48] observed a slight increase in intensity for both lines with increasing He pressures in the range of 760 Torr to 2,300 Torr. It was also observed that the ratio of the Al(II) at 281.6 nm and Al(I) at 308.2 nm emission intensity increased with increasing He pressure in the range of 760 Torr to 2,300 Torr. Owens and Majida argue that since the emission transition energy of excited He and Al(II) at 281.6 nm are close in value, energy can be transferred between He and Al(II). As the pressure increases, so does the frequency of collisions between He and Al, which results in increased emission of Al(II) at 281.6 nm line [48]. This study was conducted in a narrow pressure range and is consistent with Figure 7 from Vors *et al.* [47] where there is a slight increase in intensity observed when the He pressure is increased above 1 atm. However, pressures near 10 atm and above result in rapid reduction in emission intensity with increasing pressure as seen in Figure 7.

## 3. Influence of Atmospheric Composition (e.g., He, N_2_, Ar & CO_2_)

**Figure 9.**
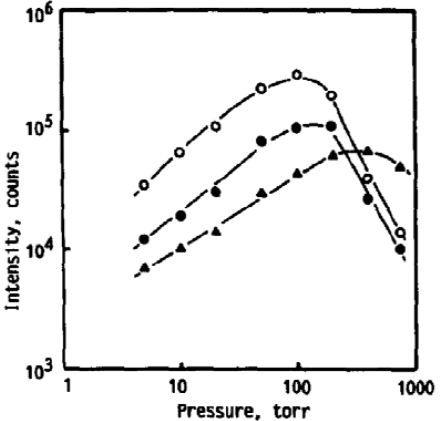
LIBS emission intensities of Fe at different atmospheric pressures, Ar (◯), air (●), and He (▴). Reprinted from reference [49].

**Figure 10.**
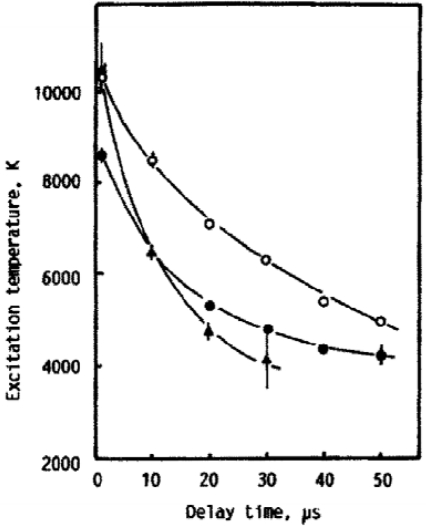
Temporal resolved plasma temperatures in different atmospheres at 100 Torr, Ar (◯), air (●), and He (▴). Reprinted from reference [49].

Iida [49] conducted a thorough study on the effect atmospheric Ar, He, and air have on the emission intensity, plasma temperature, electron density, and mass removal during LIBS. Iron emission intensity was nearly 10-fold greater under He at 1 atm compared to Ar and air at 1 atm. As pressures were reduced to 100 Torr, an atmosphere of Ar resulted in a 10-fold increase in emission intensity, air resulted nearly a 3-fold increase in emission intensity, while He resulted a slight decline in emission intensity compared to 1 atm. These trends in emission intensity *versus* atmospheric composition can be seen in Figure 9. These sets of experiments used a very long gate width of 1 ms, which should collect all meaningful emission; however, this may not be the optimum gating for LIBS experiments. It was also found in temporal studies that the plasma temperature decreased more rapidly under 100 Torr atmosphere of He compared to Ar or air, which can be seen in Figure 10. Iida also calculated approximately an order of magnitude lower LIBS plasma electron density in a He atmosphere compared to Ar at 100 Torr and 760 Torr. Also, the electron density was approximately 3 to 4 times lower at 100 Torr compared to 760 Torr for both He and Ar. Spectra taken in He from the temporal study also appeared to be of significantly higher resolution and better S/N than the spectra taken in Ar; however, Iida did not comment of this resolution difference. In addition, spectra at 100 Torr compared to 760 Torr appeared to have better S/N and higher resolution. The line width in LIBS spectra is primarily due to the Stark and Doppler broadening. Stark broadening is primarily caused from collisions between electrons and atom, and Doppler broadening is proportional to the plasma temperature. The higher resolution spectra at lower pressures and in He reported by Iida *et al*. are likely a result of a combination of lower plasma temperatures and lower electron densities. Iida also studied laser vaporization rates in He and Ar. Sensitive sample mass measurements, made during LIBS under atmospheres ranging from just under 10 to 1,000 Torr, showed a significant decrease in vaporization rates for Ar with increasing pressure. Helium experiments showed only a modest decrease in vaporization rates from 10 to 1,000 Torr. The above observations, explained by Iida, are primarily related to plasma shielding and how different gases effect plasma shielding. Laser plasmas developing rapidly with a sufficient amount of electrons can absorb a significant portion of the laser pulse through inverse Bremsstahlung. Ar is more easily ionized than He and the breakdown threshold in gas for He at 1 atm is ∼3 times greater than Ar and ∼5 times greater at 100 Torr [50]. Iida notes that the breakdown threshold is significantly less during LIBS on a surface. The lower breakdown threshold in Ar, compared to He, creates an environment favorable for plasma shielding, which will reduce vaporization of the sample and lead to a weaker LIBS signal.

Aguilera *et al*. compared the effect that 1 atmosphere of Ar, He, and air had on plasma temperature and electron densities during LIBS of steel [51]. It was determined that atmospheric Ar resulted in the highest plasma temperature and electron density, while a He atmosphere resulted in the lowest plasma temperatures and electron density. Studying temporal data, it was also found that Ar had the slowest decay of both electron density and plasma temperature, while He had the fastest decay in both parameters. As mentioned by Vors *et al*. [47] and Lee *et al*. [52], the reason the electron density and plasma temperature decay more rapidly in He compared to Ar is because He has a higher thermal conductivity than Ar.

The composition of the background pressure has been shown to greatly influence the rate of ablation. In a study by Mao *et al*. [53] involving laser ablation of copper, it was found that ablation was greater under a He atmosphere compared to an Ar atmosphere [53]. Ablation craters were twice as deep and wider in He compared to those generated in an Ar atmosphere, but the intensity of copper emission measured by ICP-AES of the ablation plume was significantly greater (16.4-fold) when using a 1,064 nm laser with a 35 ps pulse duration. The effect of using helium compared to Ar is not as impressive when a 266 nm laser with a 35 ps pulse duration (3.3-fold) or a 248 nm laser with a 30 ns pulse duration. Mao *et al*. argue that plasma shielding is likely the dominant mechanism for the improvement seen in ablation using an atmosphere of He compared to Ar. The ionization potential for He and Ar are 24.4 and 15.8 eV, respectively. Under an Ar environment, it is likely that plasma shielding could be a factor because the ionization potential is lower and ionization cross section greater than the ionization potential and ionization cross section for He.

**Figure 11.**
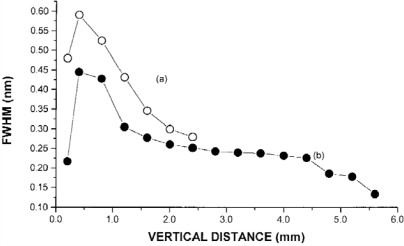
Full width at half-maximum of copper line at 521.82 from LIBS *versus* emission collection from sample surface. Reprinted from reference [52].

**Figure 12.**
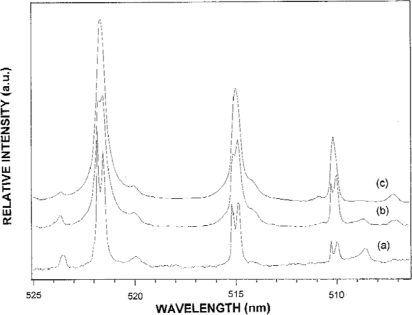
LIBS spectra of Cu in different atmospheres: **(a)** He, **(b)** Ne, and **(c)** Ar. Reprinted from reference [52].

Lee *et al*. [52] studied the influence Ar, Ne, and He as a surrounding atmosphere have on pressure broadening and self-absorption in LIBS Cu spectra. Figure 11 plots the FWHM of Cu at 521.82 nm *versus* measurement locations above the sample surface. In Figure 11a, the Cu line is broadening significantly at locations close to the sample surface regardless of whether Ar or He is the surrounding atmosphere, which is due to the high pressure and temperature near the surface. At location further away from the sample surface, the line broadening decreases; however, Cu experiences more line broadening under an atmosphere of Ar than observed in He. Because Ar is more easily ionized than He, LIBS plasmas in Ar experience greater electron density than plasmas in He. Lee *et al*. argues that increased electron density results in an increase in line broadening observed in Ar. Figure 12 compares LIBS spectra of Cu in atmospheres of Ar, Ne, and He measured 0.4 mm above the sample surface. In Figure 12 a dip in the central frequency of the three Cu lines in the He atmosphere is evidence of self-absorption. Slight self-absorption is also observed in the Ne atmosphere and no self-absorption is observed in the Ar atmosphere. Lee *et al*. also made similar measurements at 100 Torr where Ar also exhibited self-absorption. Lee *et al*. explains the results at 100 Torr by suggesting that the atmosphere acts to dampen free expansion of propelled atoms from the sample surface. Lee *et al*. also notes that, due to the greater thermal conductivity, He will cool a plasma more quickly through collisions than Ar. Though not mentioned by Lee *et al*., it may be possible that LIBS surface plasmas generated in He atmosphere or in low pressure Ar atmosphere are not as optically thin as plasmas generated at 1 atm. Compared to Ar and atmospheric conditions, an expanding LIBS plasma is less likely to breakdown the surrounding low pressure or He atmosphere. This reduced breakdown may result in a less confined plasma, and thus a larger plasma than a plasma generated in 1 atm Ar.

**Figure 13.**
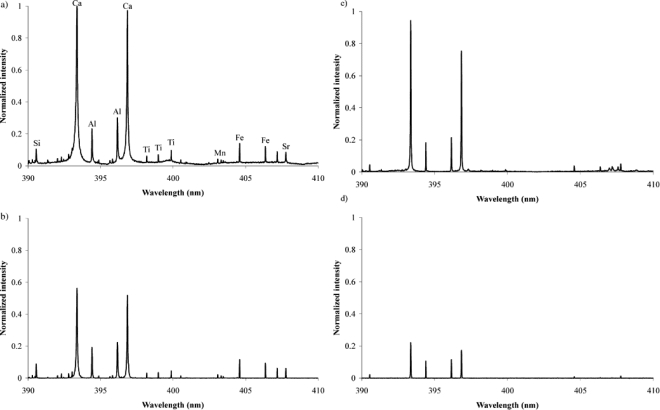
LIBS spectra of a soil. **(a)** in air no gate delay; **(b)** in air with gate delay of 1 μs; **(c)** in 7 Torr CO_2_ with no gate delay; **(d)** in 7 Torr CO_2_ with gate delay of 1 μs. Reprinted from reference [15].

The potential use of LIBS on Mars has commenced many studies involving LIBS in primarily a CO_2_ environment [15,18–24]. In Figure 13, Salle *et al*. [15] compares LIBS of soil taken with and with out gated delay and in air at atmospheric pressures and at 7 Torr CO_2_. From Figure 13, it can be seen that gating the spectrometer results in an improvement in spectral resolution and S/N; however, there is also a slight decrease in emission intensity. When comparing air at atmospheric conditions with a 7 Torr CO_2_ atmosphere, LIBS spectra exhibit improved resolution and an increase S/N for the stronger lines in a 7 Torr CO_2_ atmosphere. For example, when using a gated delay spectral lines from Al (396.15 nm) and Ca (393.37 nm) both have an improvement of around 10-fold in S/N and Si (390.56 nm) has an improvement of nearly 40-fold in S/N in a 7 Torr CO_2_ atmosphere. The intensity is weaker in 7 Torr CO_2_ and some of the weaker lines seen in the atmospheric conditions are undetectable in a 7 Torr CO_2_ atmosphere. Some of these undetectable lines are Ti (398.97 nm) and Mn (403.31 nm). Also, the S/N for Fe (404.59 nm) at 7 Torr CO_2_ was about half of the S/N at atmospheric conditions. Salle *et al*. measured the full width at half maximum of several lines between 390 to 410 nm and the majority of the lines in this region either had no or only a modest improvement in resolutions. The scope of this study was to compare calibration curves from soil and clays at atmospheric conditions (585 Torr), Mars conditions (7 Torr CO_2_) and Moon/asteroid conditions (50 mTorr air). It was found that the best repeatability and linear regression was achieved at 7 Torr CO_2_.

In another study involving Mars atmosphere simulation, Brennetot *et al*. [19] found that LIBS of basalt sample at 7 Torr CO_2_ resulted in an improvement when compared with atmospheric conditions. Brennetot *et al*. also studied the effect of LIBS at varying CO_2_ pressures; at very low pressures (<3 Torr) the plasma plume was highly expanded and difficult to fully image on to fiber optic. At pressures between 3 and 15 Torr, the lifetime of Cu (515.324 nm) decreased gradually with increasing pressures. This reduction in lifetime with increasing pressure is likely do to the increase in collisions at higher pressures, resulting in more rapid cooling of the plasma. There was no mention of whether the increase in collisions at the higher pressures resulted in line broadening; however, spectra at atmospheric conditions did exhibit significant self-adsorption compared to spectra in 7 Torr CO_2_.

Colao *et al*. [20] studied the parameters to optimize LIBS spectra in a 6 Torr CO_2_ environment. It was found that the optimum delays for LIBS at atmospheric conditions and 6 Torr CO_2_ were slightly different and that the intensity was always higher at atmospheric conditions. Although the maximum intensity is twice as intense at atmospheric conditions compared to 6 Torr CO_2_ at the respective optimum gate widths and delays, the S/N is not significantly different. This result differs from Brennetot *et al*. [19], where emission intensity is greater in 7 Torr CO_2_ compared to atmospheric conditions (*i.e.*, air at 760 Torr). This difference highlights the difficultly in comparing different LIBS experiments. Both authors note that changes in LIBS plume geometry could affect the collection of emission.

**Figure 14.**
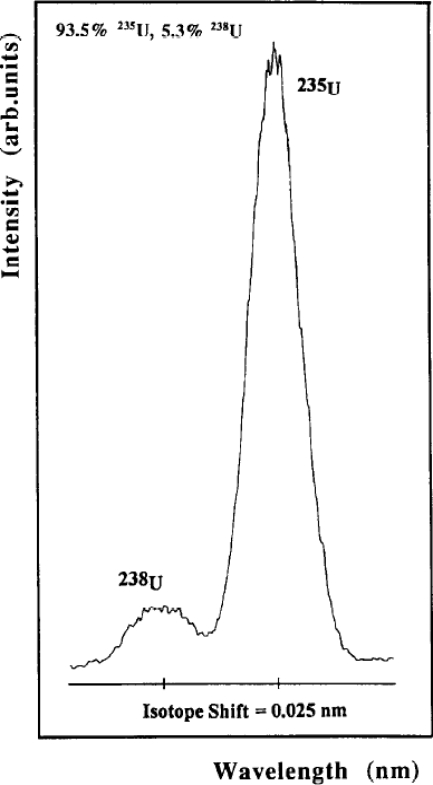
LIBS spectra of ^235^U (93.5%) and ^238^U (5.3%) Reprinted from reference [32].

LIBS requires little to no sample preparation, consumes minute quantities of material, and can be used in standoff applications. These attributes make it well suited for measurements of radioactive material. Measurements of radioactive isotope shifts require high-resolution spectroscopy; thus, LIBS is not typically used. However; Pietsch *et al*. [32] took advantage of a low pressure environment to improve spectral resolution of LIBS for U isotope measurements. Using an atmosphere of air at 2.67 Pa (∼0.02 Torr), Figure 14 illustrates the ability of LIBS at low pressure to resolve ^238^U and ^235^U, which are only 0.025 nm apart, in an enriched U sample. Pietsch *et al*. noted that to achieve lines narrow enough to separate U isotopes, LIBS must be conducted at low pressures to reduce the effects Stark broadening.

**Figure 15.**
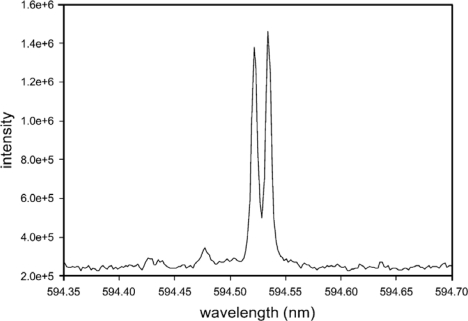
LIBS spectrum of plutonium oxide ^239^Pu (49%) and ^240^Pu (51%). Reprinted from reference [30].

In another study examining LIBS isotope measurements, Smith *et al*. [30] used a 13.3 kPa (∼100 Torr) He atmosphere to measure Pu isotopes. Figure 15 is a LIBS spectrum of plutonium oxide with a ^239^Pu/^240^Pu isotope ratio of 49/51%, respectively. Here the expected isotope shift of ^239^Pu and ^240^Pu is 0.355 cm^−1^ (0.0125 nm), and is fairly well resolved in the LIBS spectrum. Smith *et al*. used a much higher atmospheric pressure (∼100 Torr) compared to the conditions Pietsch *et al*. used (∼0.02 Torr) and explained that the higher pressure should cool the plasma more quickly leading to reduced Doppler and Stark broadening. Due to the high ionization potential of He, a pressure of 100 Torr would likely not have the same adverse effects associated with other gases, such as N_2_ or Ar, which would breakdown more easily from the expanding plasma shock wave and shield the sample.

## 4. Conclusions

Not all of the reviewed studies corroborate well with each other, in fact. the cited work by Vadillo *et al*. [41] seems to contradict results from many other experiments [15,19,39,45,49] because of the difference in experimental conditions. Many variables can affect LIBS signal, for example, emission collection optics, irradiance, gating delay and width and sample surface conditions. Brennetot *et al*. [19] noted that if a plasma plume gets too large, in low pressure atmospheres for example, light may not be completely collected if proper optical considerations are not made. Also, irradiance may play a role and contribute to the variation between experiments. Another parameter that makes it difficult to compare data is that in all experiments the gate delay and width differ, as seen in comparing Brenneton *et al*. [19] and Colao *et al*. [20]. Also, the sample composition and surface condition may also affect LIBS spectra. For example, craters at the ablation site may have a significant effect on the LIBS intensity [42–44].

Although there is no definitive study that fully accounts for all of the phenomena that occur in LIBS as the pressure and atmospheric compositions are changed, the work of Iida [49] does provide a well-rounded overview that is consistent with most other studies. It must be kept in mind that many of the reviewed studies were performed to meet specific analytical challenges, such as emulating conditions on Mars, and have demonstrated the applicability of LIBS to various atmospheric conditions.

Based on this review, it is clear that reduced pressures (<760 Torr) tend to improve LIBS spectra by increasing the S/N and improving resolution. The observed improvement is primarily due to the reduced plasma shielding, resulting in more ablation, and less Stark broadening. However, if the pressures are reduced too much (<10 Torr), then LIBS spectra tend to degrade, primarily because of lack of plasma confinement. Due to its high ionization potential, He may be useful in improving LIBS spectra when pressures cannot be reduced. The more the nuances of what occurs in the LIBS plasma are understood, the easier it will be to optimize LIBS for extreme environments and applications requiring high resolution and S/N.
